# Maternal death review and outcomes: An assessment in Lagos State, Nigeria

**DOI:** 10.1371/journal.pone.0188392

**Published:** 2017-12-14

**Authors:** Friday Okonofua, Donald Imosemi, Brian Igboin, Adegboyega Adeyemi, Chioma Chibuko, Adewale Idowu, Wilson Imongan

**Affiliations:** 1 Program and Research Unit, Women’s Health and Action Research Centre (WHARC), Benin City, Edo State, Nigeria; 2 University of Medical Sciences (UNIMED), Ondo, Ondo State, Nigeria; 3 Centre of Excellence in Reproductive Health Innovation (CERHI), University of Benin, Benin City, Edo State, Nigeria; 4 Department of Obstetrics and Gynecology, Lagos Island Maternity Hospital (LIMH), Lagos State, Nigeria; 5 Department of Obstetrics and Gynecology, Gbagada General Hospital (GGH), Lagos State, Nigeria; 6 Department of Obstetrics and Gynecology, Ajeromi General Hospital (AGH), Lagos State, Nigeria; National Academy of Medical Sciences, NEPAL

## Abstract

The objective of the study was to investigate the results of Maternal and Perinatal Death Surveillance and Response (MPDSR) conducted in three referral hospitals in Lagos State, Nigeria over a two-year period and to report the outcomes and the lessons learned. MPDRS panels were constituted in the three hospitals, and beginning from January 2015, we conducted monthly MPDSR in the three hospitals using a nationally approved protocol. Data on births and deaths and causes of deaths as identified by the MPDSR panels were collated in the hospitals. The results show that over a 21-month period (January 1, 2015 –September 30, 2016), maternal mortality ratio (MMR) remained high in the hospitals. Although there was a trend towards an increase in MMR in Lagos Island Maternity Hospital and Gbagada General Hospital, and a trend towards a decline in Ajeromi Hospital, none of these trends were statistically significant. Eclampsia, primary post-partum haemorrhage, obstructed labour and puerperal sepsis were the leading obstetric causes of death. By contrast, delay in arrival in hospital, the lack of antenatal care and patients’ refusal to receive recommended treatment were the patients’ associated causes of death, while delay in treatment, poor use of treatment protocols, lack of equipment and lack of skills by providers to use available equipment were the identified facility-related causes of death. Failure to address the patients and facility-related causes of maternal mortality possibly accounted for the persistently high maternal mortality ratio in the hospitals. We conclude that interventions aimed at redressing all causes of maternal deaths identified in the reviews will likely reduce the maternal mortality ratios in the hospitals.

## Introduction

The high rate of maternal mortality in Nigeria has been a major cause of public health concern at both national and international levels. In response to recommendations made by experts. [1] and in efforts to address this developmental challenge, Nigeria’s Federal Ministry of Health in 2013 approved that all maternal health institutions in the country should periodically carry out maternal death review, surveillance and response [2], using the technical guidance document as recommended by the WHO [3]. This was updated in 2016 to include Perinatal Death Reviews, while the initiative was retitled Maternal and Perinatal Death Surveillance and Response (MPDSR), to take account of the equally high rates of stillbirth, neonatal and perinatal deaths in the country [4]. Essentially, it is hoped that with regular reviews of maternal and perinatal deaths, and an analysis of the causes of deaths, that recommendations could be made that if addressed, would reduce the high rates of maternal and perinatal mortality in the country. This approach was required to address the current lack of substantive data on the circumstances under which maternal and perinatal deaths occur in Nigeria, needed to design strategies, policies and programmes at a health systems level to reverse the trend.

Since its promulgation, the Federal Ministry of Health has recommended that all States in the country adopt a uniform protocol for conducting the reviews [5]. The Lagos State Ministry of Health, one of Nigeria’s 36 federating States, started implementing the recommendations and protocol in 2014. This included an initial state-wide assessment of maternal mortality rates which identified un-acceptably high rates in the most rural Local Government Areas of the State [6,7]. Thereafter, State officials constituted a Committee to review maternal deaths using the methods and protocols recommended by the Federal Ministry of Health [8]. The Committee was given the mandate to collate accurate data and make recommendations on ways to reduce the rate of maternal deaths in the State.

Our initial systematic analysis of maternal death reviews in Nigeria suggests that substantial information can be obtained through this process to strengthen health systems in efforts to reduce maternal mortality rates [9, 10]. Since previous maternal death reviews were not systematically carried out in many parts of the country, we sought to follow up reviews conducted in three public hospitals in Lagos state in a prospective manner in order to report the outcomes and the lessons learnt. In particular, as this review added the element of surveillance and policy/programmatic feedback, we sought to determine how this approach might impact on maternal mortality prevention. We hypothesized that maternal mortality would decline if corrective measures are taken to rectify the medical and social factors that are identified in the reports as associated with maternal mortality. The objective of this study therefore, is to report the results and outcomes of maternal death reviews conducted in the hospitals over the initial two-year period. We believe the results will be useful for improving the delivery of maternal death reviews and surveillance, and eventually provide useful lessons for scaling up the method throughout the country.

## Methods

The study was conducted by researchers from the Women’s Health and Action Research Centre (WHARC), a leading non-governmental, not-for-profit organization in Nigeria whose mission is to promote the health and social well-being of women through research, documentation and advocacy. In efforts to implement the study, WHARC met with officials of the Lagos State Ministry of Health and its Committee on Maternal and Child Mortality Reduction Programme (MCMR). The study objectives were explained, which led the officials of the State to give permission for the research team to monitor maternal deaths under the MPDSR conducted in three of its public maternity hospitals. The hospitals were: Ajeromi General Hospital (AGH), Gbagada General Hospital (GGH), and the Lagos Island Maternity Hospital (LIMH) in Lagos Island. All three offer routine antenatal, delivery and postnatal care as well as comprehensive emergency obstetrics care to pregnant women–covering very large populations in Lagos State.

Before commencing the project, we sought permission to conduct the study from the Management of the hospitals after a full explanation of the purpose and methods of the project. Thereafter, we conducted workshops in the hospitals to create awareness of the study and to increase understanding of the MPDSR process. We also met individually with members of the MPDSR committees in the three hospitals to explain the objectives of the study and also to develop a shared understanding of the MPDSR process. The workshops emphasized the importance of using confidential approaches, including a no-blame enquiry in the conduct of the reviews, in consonance with the recommendations of the FMOH. The committees gave permission for a member of the research team to be present at the committee meetings as an observer without any rights to make submissions at the meetings. They were assured of confidentiality of any information obtained, and no names of specific patients were presented during the committee meetings and thereafter.

Thereafter, WHARC supported the organization of the meetings of the MPDSR committees in the hospitals, which held monthly. This report presents the results of the monthly MPDSR committee meetings held in the three hospitals over 21 months from January 2015 to September 2016. The data were then cross-checked with available data in the Medical Records Departments to ensure accuracy.

A report and an analysis of the methodology used in the committee meetings has been documented elsewhere [10]. In brief, MPDSR was conducted in a structured way and had multidisciplinary representation. We grouped discursive strategies observed into three overlapping clusters: ‘doing’ no-name no-blame; fostering participation; and managing personal accountability. Within these clusters, explicit reminders, gentle enquiries and instilling a sense of togetherness were used in doing a no-name, no-blame enquiry.

As recommended by the FMOH, the committees consisted of the Chairman of the Medical Advisory Committee/Director of Clinical Services as Chairperson, while the Head of the Department of Obstetrics and Gynaecology is Secretary. Other members were Heads of Departments of Nursing/Midwifery, Paediatrics, Pathology, Labour ward, Neonatal care, anaesthesia and haematology.

## Data collection and analysis

We collected data on the number of deliveries during the month, the number of maternal deaths, and the proportion of women who died that received antenatal care in the same hospital (booked cases) versus those who did not receive antenatal care (unbooked cases). We then calculated the maternal mortality ratios (MMR) (number of maternal deaths per 100,000 live births) by month per hospital, and calculated the overall MMR for the total cohort of women. We compared ratios among the hospitals, developed monthly trends and compared the monthly differences using the chi-square (χ^2^) test for trends. We then adjusted the analysis for confounders such as increase or decrease in number of deliveries, and proportion of complicated deliveries in relation to the decrease or increase in MMR.

Following the reviews in each hospital, we identified the medical causes as well as the social (background) causes associated with the deaths. Each committee made recommendations to the heads of the institutions and also to the State government for rectifying the identified causes of deaths. We compiled these recommendations for each hospital, and analyzed qualitatively for form, theme and content. The results were presented qualitatively for each hospital and overall, to identify the nature of recommendations made for averting maternal mortality in the hospitals.

Ethical approval for the study was obtained from the Lagos State Ethical Review Board, and also consent was obtained from the Lagos State Ministry of Health and the individual health facilities. Additionally, all data were anonymized before access to the researchers and the MPDSR committees.

## Results

### Maternal Mortality Ratios (MMR)

The results presented in Table 1 show that there were a total of 164 maternal deaths in the three hospitals during the period, and a total of 10,237 live births, giving an overall MMR of 1,602/100,000 live births. As shown, the LIMH had the highest MMR, while GH had the lowest.

**Table 1:** Maternal deaths and Maternal Mortality Ratios (MMR) by health facilities.

Name of health facility	Total number of MPDSR	Total number of deliveries	Total number of maternal deaths	MMR = maternal deaths/total number of deliveries x 100,000
Ajeromi Hospital	21	1812	19	1048.6/100,000
Gbagada Hospital	21	2939	29	986.7/100,000
Lagos Island Maternity Hospital	21	5486	116	2114.5/100,000
Total	63	10237	164	1602/100,000

The results in Table 2 show that “unbooked” women (women who had not received antenatal care in the hospital, but who presented as emergencies with complicated labour) accounted for a high proportion of the maternal deaths in the three hospitals. Nearly 90% of the deaths in both LIMH and AGH occurred in “unbooked women”, while about 79.0% occurred at the GH. By contrast, fewer proportions of deaths were reported in “booked” women–these were women who reported for antenatal care and delivered in the same hospitals.

**Table 2:** Maternal deaths by hospital booking status.

Hospital	Number of maternal deaths, unbooked	Number of maternal deaths, booked	Total maternal deaths	Unbooked women as % of maternal deaths
Ajeromi General Hospital	17	2	19	89.5
Ggagada General Hospital	23	6	29	79.3
Lagos Island Maternity Hospital	104	12	116	89.7
Total	144	20	164	87.8

### Trends in Maternal Mortality Ratio

**Fig 1:**
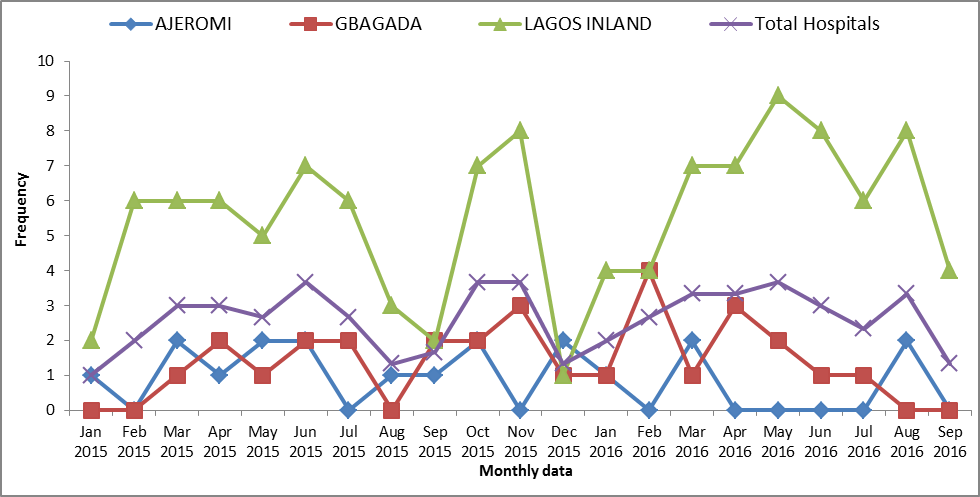
Trends in Maternal Mortality ratio by months in the three hospitals and overall trends. A wide variation in maternal mortality ratio was observed among the three Hospital in 21-month period.

**Fig 2:**
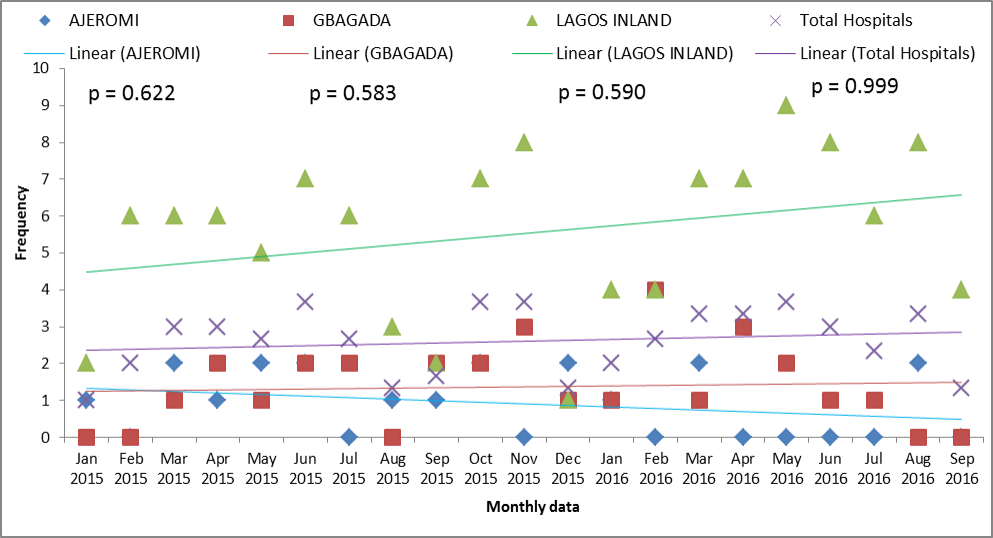
Trends plot of maternal mortality in Lagos state. An increase in MMR in LIMH (χ^2^ = 17.97; df = 20; p = 0.590), in GGH ((χ^2^ = 18.07; df = 20; p = 0.583) while a decrease in MMR was observed in AGH (χ^2^ = 17.47; df = 20 p = 0.622). However, this observation was not significant at p<0.05.

The monthly trends in MMR over the 21 months’ period in the 3 hospitals and overall are plotted in Fig 1. There was wide variation in the trends in the hospitals, but as shown in Fig 2, we plotted a best fit linear trend in each hospital and overall. The results show a trend towards an increase in MMR in LIMH and GGH, and a trend towards a decrease in MMR in AGH. Overall, there was a slight trend towards an increase in MMR in the three hospitals. However, the chi-square test for trends at 95% significance level, was not statistically significant in the pattern observed for AGH (χ^2^ = 17.47; df = 20 p = 0.622), GGH (χ^2^ = 18.07; df = 20; p = 0.583); LIMH (χ^2^ = 17.97; df = 20; p = 0.590) and the overall hospitals (χ^2^ = 6.03; df = 20; p = 0.999).

### Obstetric causes of maternal deaths

The medical and obstetric causes of deaths as reported in the MPDRS conducted in the three hospitals are presented in Table 3. As shown, eclampsia, primary postpartum haemorrhage, prolonged obstructed labour, maternal sepsis and antepartum hemorrhage were the leading obstetric causes of maternal mortality in the hospitals. Medical complications–cardiac failure, sickle cell disease and malaria–were unusually high as causes of maternal deaths, especially at the LIMH.

**Table 3:** Medical causes (primary causes) of maternal deaths by health facilities.

Primary Causes of Maternal Death	Ajeromi Hospital	Gbagada Hospital	Lagos Island Maternity Hospital	Total
Eclampsia	3	8	36	47
Post-partum Hemorrhage	2	4	27	33
Prolonged Obstructed Labour	4	3	4	11
Sepsis	1	4	5	10
Uterine Rupture	1	2	4	7
Ante-Partum Hemorrhage	1	1	8	10
Ectopic gestation	0	0	2	2
Anaemia in Pregnancy	1	1	2	4
Sickle cell disease	2	0	3	5
Cardiac failure	4	2	5	11
Malaria	0	0	1	1
HIV/AIDS	0	0	5	5
Dead on arrival	0	0	5	5
Others	0	4	9	13
**Total**	**19**	**29**	**116**	**164**

**Others:** Thromboembolism (3), Acute renal failure (2), Disseminated intravascular coagulopathy (2), hypovolemic shock (1), Meningitis (3) infection (2)

### Associated causes of maternal deaths

The MPDSR in the three hospitals were designed to establish the associated causes of maternal mortality, especially the background social factors associated with maternal deaths in the hospitals. The results of the associated causes of death elicited from the MPDSR are shown in Table 4. Delay in presentation in the hospital (Type 1 Delay) [11] ranked high in the three hospitals as epitomized by results such as “delayed presentation in health facility”, “lack of antenatal care services”, “patient’s refusal of prompt management at admission” and “no evidence of antenatal services”. These were patients’ related factors but there was also reports that some patients received “inadequate/wrong care from traditional birth attendants” or as reported from GH and the LIMH, that some patients were mismanaged in private hospitals from where they were referred.

**Table 4:** The major identified associated causes of maternal deaths in the hospitals.

Associated causes of Maternal death	Ajeromi Hospital	Gbagada Hospital	Lagos Island Maternity Hosp	Total
Delayed presentation to facility	6	4	47	**57**
Delay in patient management	1	3	6	**10**
Non-availability of intensive care unit (ICU)	2	3	0	**5**
Non-availability of blood product when needed	3	4	6	**13**
Poor/inadequate antenatal care	1	3	6	**10**
No evidence of antenatal care	3	0	12	**15**
Poor compliance with treatment	1	3	8	**12**
Lack of diagnostic procedures	0	2	4	**6**
Patient refusal of treatment on admission	1	3	11	**15**
Poor case mismanagement from referral facility (TBAs/private facility etc.)	1	3	14	**18**
Lack of skills to use essential emergency equipment	0	1	2	**3**
Total	19	29	116	**164**

Many reports of poor or delayed management of the cases (Type 3 delay) in the three hospitals also featured in the MPDSR reports. As shown in Table 4, these included “non-availability of blood” in the three hospitals, “non-availability of intensive care unit” “poor compliance to treatment protocols”, “delay in patient management”, “lack of essential equipment”, and the “lack of skills by staff to use available equipment, e.g. anti-shock garments”, etc.

### Recommendations by MPDSR Committees

The recommendations made for rectifying the associated causes of maternal mortality in the three hospitals are presented in Table 5. The recommendations were largely derived from the deficits identified and included community health education, training of staff to better use treatment protocols, provision/repair of equipment, provision of emergency obstetric care and intensive care among several others. However, there was no clear evidence that these recommendations which went to policy makers and hospital administrators were fully implemented by any of the hospitals during the period.

**Table 5:** Recommendations on preventing maternal deaths by health facilities.

Ajeromi Hospital	Gbagada Hospital	Lagos Island Maternity Hosp
1. Provision of intensive care unit for postpartum complication management 2. Competent ANC skilled attendant at birth 3. Community education 4. Provision blood product 5. Presence of skilled attendant at birth to recognize PPH 6. Make avail emergency kits 7. Make avail resuscitating materials and anaesthesia equipment 8. Make available more ambulance	1. Provision of an intensive care unit that meets international standard. 2. Urgent provisions of monitors for effective management of patients 3. Training of senior nursing personnel on basic lifesaving skills 4. Expansion of the blood bank system for more availability of blood 5. Readily availability of blood product 6. Government develop policies for TBAs 7. Continuous training of all health care personnel by the hospital management 8. Provision of AC to cool COBAS machine used for clinical biochemistry 9. Recording vital sign as mere routine duty should be discontinued 10. MDR committee to implore PHC not to take delivery for primigravida due to associated risk 11. Continuous training of TBAs 12. Provision of more space and manpower in stemming the fight of maternal mortalities by the state government	1. Expansion of intensive care unit 2. Equip the ICU 3. Proper supervision of TBAs, mission homes by relevant bodies such as Health Facilities Accreditation and Monitoring Agency 4. To ensure maintenance and improvement of standard of care in facility 5. Ensure availability of blood 6. Public awareness, education through campaigns in market places, community using fliers radio jingles billboards and involvement of community leaders on importance of i. ANC, ii. Consulting health facility on time, iii. Delivering at a health facility iv. Complying with medical advice and treatment 7. Training of staff on i. Resuscitation procedure ii. On emergency obstetric care 8. Increase man power in the facility 9. Reinforce communication between facilities to refer patients with complication on time 10. Refer patient within critical condition to the ICU on time 11. Prioritization of investigations must always be done in view of patients finances

## Discussion

The study was designed to investigate the results of maternal death reviews in three hospitals in Lagos State, a leading Nigerian State that commenced the nationally recommended guidelines for MPDSR. This assessment also enabled us to collate data and statistics on MMR accurately in a prospective manner. To the best of our knowledge, this is one of a few prospective studies ever carried out in the country to obtain accurate data on MMR. The results showed very high MMR in the three hospitals, which are consistent with reports from various hospitals in the country. The LIMH had the highest ratio of 2114.5/100,000 live births, while the GH had the lowest ratio of 986.7/100,000 live births. Overall, the results show figures that are much higher than recently reported national community estimates of maternal deaths [12,13, 14], which is probably due to the fact that the three hospitals are referral hospitals that receive women as obstetric emergencies from lower levels of health care provisions. However, it may also be due to a true increase in MMR in the hospitals.

The objective of the national guideline for MPDSR is to identify the medical and associated causes of maternal mortality in maternity hospitals in a “no-blame manner”, and to correct the deficiencies that lead to death in order to prevent future deaths in the hospitals. Thus, we hypothesized that if the correct measures are taken, MMR would systematically decline in the three hospitals over the months of implementation of the review. However, contrary to our expectation, MMR did not decline in any of the hospitals. Although there was a trend towards a slight decline in MMR at Ajeromi Hospital, there were also trends towards some increases in the LIMH and the GH. Although none of these trends were statistically significant, the results suggest the lack of effectiveness of the MPDSR process in preventing maternal mortality in the hospitals. We believe this to be due to the fact that although the medical and associated causes of MMR were identified in the hospitals, there was no evidence that the reported deficits were corrected at least on the short term. Future efforts to correct these deficiencies would hopefully lead to a true sustainable decline in MMR in the hospitals.

The results show that eclampsia, primary post-partum haemorrhage, prolonged obstructed labour and sepsis were the leading obstetric causes of maternal mortality in the three hospitals. These are consistent with reports from various hospitals in the country [15, 16, 17]. Among the associated factors identified by the MPDSR as responsible for deaths in women with these complications, patients’ factors topped the list. These included the non-use of antenatal care, use of alternative providers such as traditional birth attendants, late presentation in hospital, and refusal by patients to receive the recommended methods of treatment. Indeed, among the women who died from these complications, more that 80% were women who had not received antenatal care, but who presented late in hospitals when complications occurred. This is consistent with Type 1 of the three-delay model previously proposed by Thaddeus and Maine [11] and is similar in pattern to previous reviews of maternal mortality from other parts of Nigeria, with late presentation in hospital, being a major determinant factor [18,19,20]. Previous studies have also identified the inability to pay for services, illiteracy, cultural beliefs [21, 22], and poor satisfaction with services in health facilities [23] as reasons that women fail to attend early antenatal and delivery care in Nigeria. Clearly, there is a need to focus on improving the quality of delivery of maternal health services in both public and private sectors as well as on patients’ education in efforts to promote the use of evidence-based pregnancy care by women. The use of safety nets such as conditional cash transfers or the elimination of user fees are examples of successful interventions that have been used to incentivize women to seek early pregnancy and emergency obstetric care in resource-poor countries [24,25]. Such measures which have been successfully used in parts of Nigeria [26] would also be useful in Lagos State to reduce the incidence of such delays. As shown in this report, some women with complications had been delayed in private hospitals before a decision was taken by care-givers in those health facilities to refer such women to higher levels of care. An additional measure therefore would be to re-train of private providers of maternity care to update their skills and knowledge, so as to ensure that they promptly refer women in difficult labour to higher levels of health care.

The study also identified various institutional factors from the MPDSR reports (Type 3 delays) that account for deaths from obstetric complications. These included delay in management of patients, non-availability of blood and blood products for transfusing haemorrhaging women, lack of equipment, inadequate skills of providers to use available equipment, non-adherence to treatment protocols by providers, and actual cases of mis-management of patients. It is noteworthy that the reviews were able to identify such institutional factors as being responsible or associated with maternal mortality. This may be due to the “no-blame method” and confidential approach adopted and the careful systematic process used for the review as previously documented [10]. Efforts to correct these institutional deficits should include the training and re-training of health providers, the development of institutional policies and guidelines for provision of maternal health care, provision of relevant equipment and training of staff to use them, and regular provision of blood and blood products. In particular, treatment protocols and guidelines should target the leading causes of maternal mortality, with emphasis placed on building staff competencies and agencies to handle them effectively. Additionally, equipment provision and infrastructural improvements have to be given top priority by the government and hospital management authorities to correct the related deficiencies that were identified in this report.

To the best of our knowledge, this is the first description of the results of the nationally adopted MPDSR process from any part of Nigeria. The strength of the study lies in the prospective method of data collection over 21 months, and the involvement of three major hospitals in Lagos State, which allowed for institutional comparison of the data. Furthermore, the fact that the data were collected by individuals “external” to the review process engendered greater objectivity and accuracy of the results.

However, the results are limited by the fact that although various associated factors were identified as causes of maternal mortality, no substantive efforts were made to correct the deficiencies in any of the hospitals. Thus, our results could be identified as baseline results which would change significantly if efforts are made to address the patients’ related and institutional factors that lead to maternal mortality in the hospital. The next step is to develop an advocacy platform [27, 28] of action to build political will for ensuring that the recommendations made by the MPDSR Committees are implemented by the hospital management authorities and the supervising government agencies.

## Conclusion

The results of maternal death reviews in three hospitals in Lagos State of Nigeria show persistently high MMRs, and have identified the obstetric and social factors that predispose to maternal deaths in the hospitals. These factors include obstetric complications, but are heightened by the inadequate demand for services, poor delivery of services and inadequate equipment to deliver quality care in the health referral facilities. Unfortunately, there has been little evidence to show that these factors were addressed during the period of the study. We believe that efforts devoted to addressing these factors will lead to a significant reduction in maternal mortality ratios in the hospitals. In particular, strong political will by the management of the hospitals and the supervising government agencies is a prerequisite to address the human and infrastructural deficits that predispose to maternal mortality in maternity hospitals in Lagos State. Such improvements when demonstrated can be scaled up to other Nigerian States to achieve sustainable reduction in maternal mortality throughout the country.
